# A multi-year assessment of blacklegged tick (*Ixodes scapularis*) population establishment and Lyme disease risk areas in Ottawa, Canada, 2017-2019

**DOI:** 10.1371/journal.pone.0246484

**Published:** 2021-02-04

**Authors:** Holly Burrows, Benoit Talbot, Roman McKay, Andreea Slatculescu, James Logan, Charles Thickstun, L. Robbin Lindsay, Antonia Dibernardo, Jules K. Koffi, Nicholas H. Ogden, Manisha A. Kulkarni

**Affiliations:** 1 Yale School of Public Health, Yale University, New Haven, Connecticut, United States of America; 2 School of Epidemiology and Public Health, University of Ottawa, Ottawa, Ontario, Canada; 3 Zoonotic Diseases and Special Pathogens Division, National Microbiology Laboratory, Public Health Agency of Canada, Winnipeg, Manitoba, Canada; 4 Centre for Foodborne, Environmental and Zoonotic Infectious Diseases, Public Health Agency of Canada, Saint-Hyacinthe, Quebec, Canada; 5 Public Health Risk Sciences Division, National Microbiology Laboratory, Public Health Agency of Canada, Saint-Hyacinthe, Quebec, Canada; University of Kentucky College of Medicine, UNITED STATES

## Abstract

Canadians face an emerging threat of Lyme disease due to the northward expansion of the tick vector, *Ixodes scapularis*. We evaluated the degree of *I*. *scapularis* population establishment and *Borrelia burgdorferi* occurrence in the city of Ottawa, Ontario, Canada from 2017–2019 using active surveillance at 28 sites. We used a field indicator tool developed by Clow et al. to determine the risk of *I*. *scapularis* establishment for each tick cohort at each site using the results of drag sampling. Based on results obtained with the field indicator tool, we assigned each site an ecological classification describing the pattern of tick colonization over two successive cohorts (cohort 1 was comprised of ticks collected in fall 2017 and spring 2018, and cohort 2 was collected in fall 2018 and spring 2019). Total annual site-specific *I*. *scapularis* density ranged from 0 to 16.3 ticks per person-hour. Sites with the highest density were located within the Greenbelt zone, in the suburban/rural areas in the western portion of the city of Ottawa, and along the Ottawa River; the lowest densities occurred at sites in the suburban/urban core. *B*. *burgdorferi* infection rates exhibited a similar spatial distribution pattern. Of the 23 sites for which data for two tick cohorts were available, 11 sites were classified as “high-stable”, 4 were classified as “emerging”, 2 were classified as “low-stable”, and 6 were classified as “non-zero”. *B*. *burgdorferi*-infected ticks were found at all high-stable sites, and at one emerging site. These findings suggest that high-stable sites pose a risk of Lyme disease exposure to the community as they have reproducing tick populations with consistent levels of *B*. *burgdorferi* infection. Continued surveillance for *I*. *scapularis*, *B*. *burgdorferi*, and range expansion of other tick species and emerging tick-borne pathogens is important to identify areas posing a high risk for human exposure to tick-borne pathogens in the face of ongoing climate change and urban expansion.

## Introduction

Canadians face an emerging risk of Lyme disease (LD) and other tick-borne pathogens due to the northward expansion of the tick vector, the blacklegged tick, *Ixodes scapularis* (sometimes called the deer tick). In the province of Ontario, a range expansion of the blacklegged tick has been observed since the 1990s and is projected to continue, driven in part by climate and land use changes [1–4]. The city of Ottawa, situated near the northern border of the current range of *I*. *scapularis* distribution, has recorded an increase in LD incidence in recent years, averaging 13, 49 and 119 cases per year for the periods 2010–2012, 2013–2015, and 2016–2019, respectively. [5, 6]. Given Ottawa’s geographic location within the climatically suitable range for *I*. *scapularis* and the recent increase in LD cases, there is a need for ongoing surveillance of *I*. *scapularis* and *Borrelia burgdorferi* sensu stricto (s.s.), the causative agent of LD in Ontario [1, 6–8].

There are two types of tick surveillance: passive and active. Passive surveillance involves the submission of ticks previously attached to people from healthcare providers and/or the public [9]. Active surveillance involves field sampling of ticks either by dragging or small mammal sampling [9]. While passive surveillance is useful for signalling the presence of a potential emerging risk area for tick-borne illness, active surveillance is required for determining whether a reproducing tick population is established [9]. Although adventitious ticks collected in passive tick surveillance represent a low but persistent risk of LD, the presence of a reproducing tick population in a given geographic area signals a higher potential risk for human LD exposure.

The dynamic nature of tick colonization and the long life cycle of blacklegged ticks requires a multi-year effort to identify locations with established populations. In eastern Ontario, *I*. *scapularis* populations tend to develop free of or with a very low prevalence (i.e., <10% in adult ticks) of infection with *B*. *burgdorferi* with an estimated 5-year lag between tick population establishment and the establishment of *B*. *burgdorferi* transmission cycles [10, 11]. This phenomenon is due to both the threshold of tick abundance required for the basic reproductive number of *B*. *burgdorferi* to exceed one and the lifecycle and seasonal activity of ticks [11, 12]. In eastern Canada, the phenology of *I*. *scapularis* is such that adult tick density peaks in the fall and subsequent spring, while nymphs are most active in the early summer months and larvae are most active the late summer [13]. A tick population is generally considered established if all three life stages are detected for two consecutive years using active surveillance methods [9]. However, since multi-year active surveillance is time and labour intensive, Clow et al. recently developed a “field indicator” tool that assigns a qualitative level of risk of tick establishment to a site based on one session of tick dragging [13]. While areas with evidence of tick population establishment that are detected via drag sampling are generally considered ‘Lyme disease risk areas’, to ascertain whether a locality is endemic for *B*. *burgdorferi* transmission there must be additional evidence of *B*. *burgdorferi* in ticks and/or reservoir hosts [14].

Timely information on Ottawa area tick populations is needed to inform public health measures, including targeted health promotion interventions designed to increase awareness and preventive measures among the public in areas with high risk for exposure to infected blacklegged ticks. There is also a need to improve *I*. *scapularis* and *B*. *burgdorferi* awareness among health care professionals, with the goal of encouraging early detection and treatment of people who develop LD. The aim of this study was to evaluate the presence and degree of establishment of blacklegged tick populations and *B*. *burgdorferi* in the city of Ottawa over a three-year period, 2017–2019. This information can guide public health action on Lyme and other tick-borne diseases, and will serve as a baseline for future monitoring of tick and tick-borne pathogen emergence in the region.

## Materials and methods

### Study site

**Fig 1:**
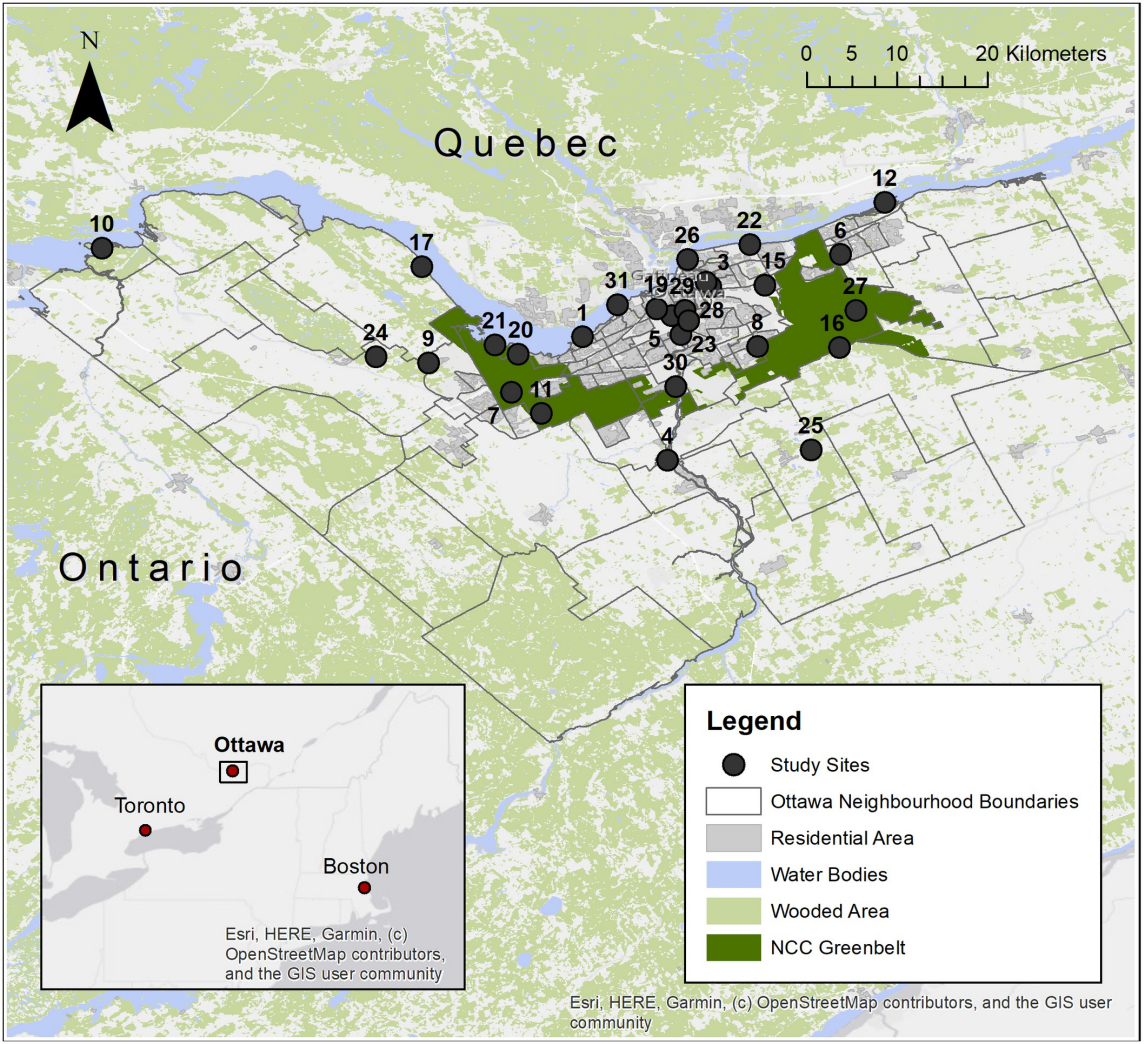
Map of 28 study sites for tick drag sampling in Ottawa, Ontario, 2017–2019.

The city of Ottawa, home to around one million people, is located on the south bank of the Ottawa river and contains a diverse set of landscapes over a geographic area of almost 3,000 km^2^ [15]. A network of trails and green space known as the Greenbelt, covering almost 15 km^2^, forms a ring around the most densely populated area of the city [16]. Twenty-eight (28) sites were randomly selected within urban, suburban, and rural strata to be spatially representative of the study region. Selected sites included parks, recreational trails, and/or conservation areas that were publicly accessible (Fig 1) [17]. Land access permits for tick sampling were obtained from the National Capital Commission and the City of Ottawa.

### Field sampling

Field sampling followed Public Health Ontario (PHO)’s active tick surveillance strategy, which recommends sampling twice per year to enable the detection of multiple tick life stages, with timing optimized to detect two adult tick cohorts in a given year [18]. Using this approach, the majority of sites were visited twice per year in 2017 and 2018 in both the spring/early summer (i.e. May to June, hereafter referred to as ‘spring’ season) and fall (i.e. late September to October); sites were only visited in the spring season in 2019. Sixteen sites were visited in spring 2017 with six sites added in the fall sampling round. An additional six sites were included in 2018 to cover a broader geographic area. Some sites were not visited in certain seasons or years due to logistical constraints and flooding. Active surveillance was conducted using the drag sampling method described by PHO [18]. Ticks were collected by dragging a 1 m^2^ cloth over the forest floor and vegetation. Sampling was conducted for at least three person-hours for larger sites, and fewer than three-person hours for small sites (minimum 1.5 person-hours). Approximately every 50 metres, the timer was stopped and the drag cloth was checked for ticks, and geographic coordinates were logged using a handheld global positioning system (GPS) device (Garmin eTrex 20x). All adult, nymph, and larval ticks were collected in plastic tubes and transported on ice to the University of Ottawa laboratory for species identification and possible testing.

### Laboratory testing

Adults, nymphs, and larvae were examined by microscopy for species and sex identification using standard taxonomic keys [19–23]. Adult and nymphal *I*. *scapularis* ticks were tested for *B*. *burgdorferi* s.s., *A*. *phagocytophilum*, and *B*. *miyamotoi* using quantitative polymerase chain reaction (qPCR) assays according to previously published protocols [17, 24]. In brief, total genomic DNA was extracted using the QIAamp mini kit (GIAGEN Inc., Mississauga, Ontario, Canada). A duplex qPCR assay targeting the 23S rRNA and the *msp2* genes was used to identify *Borrelia* species and *A*. *phagocytophilum*, respectively. In samples positive for *Borrelia*, we confirmed the presence of *B*. *burgdorferi* s.s. by targeting the *ospA* gene; *B*. *miyamotoi* confirmation targeted the *glpQ* (2017–2018) or *flaB* (2019) genes. Amplification was carried out using the BioRad CFX96 Real-Time PCR Detection System (BioRad, Mississauga, Ontario, Canada) and analysis was performed used the CFX Maestro software (BioRad, Mississauga, Ontario, Canada).

### Descriptive analyses

*I*. *scapularis* density and infection rates were calculated by calendar year, combining data from spring and fall collections, where applicable. Total *I*. *scapularis* density was calculated as the sum of all adults, nymphs, and larvae collected, divided by the number of person-hours of sampling conducted at a given site. Nymphal density was calculated as the number of nymphs collected divided by the person-hours of sampling. Infection rates were calculated as the number of *I*. *scapularis* ticks positive for *B*. *burgdorferi*, *A*. *phagocytophilum*, or *B*. *miyamotoi* divided by the total number of adult and nymphal *I*. *scapularis* tested in a given year, at a given site. Larvae were not tested for *B*. *burgdorferi* as there is no evidence of transovarial transmission of *B*. *burgdorferi* [25]. Patterns in *I*. *scapularis* density and *B*. *burgdorferi* infection rates were mapped using ArcGIS v.10.7.1 (ESRI, Redlands, USA). All map figures were created using publicly available spatial data for administrative boundaries, landuse and landcover obtained from the city of Ottawa’s GeoOttawa portal (https://maps.ottawa.ca/geoottawa/) and predefined ArcGIS basemap layers (ESRI, Redlands, USA).

### Application of risk indicator

The field indicator for risk of *I*. *scapularis* establishment was developed by Clow et al. to assist public health officials in determining the likelihood of a reproducing population of *I*. *scapularis* at a given site [13]. The indicator tool is in the form of a flow chart that takes researchers and public health officials through four questions, with a certain number of points assigned based on the answer to each [13]. Once points are assigned, a site is then allocated a qualitative risk level based on its score. In brief, a score of 0 is assigned a non-zero risk, meaning that no ticks are detected at the site; however, there remains a risk of introduction of adventitious ticks due to migratory birds [13]. A score of 1 or 2 is assigned a low risk, indicating that a single tick of any life stage was detected during the drag, or in a previous round of field sampling. A score of 3 or 4 corresponds to medium risk, indicating stronger evidence that a reproducing population is present at a low density. A score of 5 or greater is assigned high risk, suggesting strong evidence of a reproducing population of *I*. *scapularis* [13].

We applied the tool to data from a subset of 23 sites that had the most complete multi-year data to classify the likelihood of tick establishment in each site and assess trends over time. Due to the multi-year life cycle of *I*. *scapularis* ticks, we combined data on relevant tick life stages from successive fall and spring sampling periods to create discrete *I*. *scapularis* cohorts. In northeastern North America, adult and larval *I*. *scapularis* that are active in the spring tend to be individuals that did not successfully feed in the previous summer or fall, and overwintered unfed [13]. Therefore, adults and larvae collected in the spring are likely from the same cohort as any adults and larvae collected in the previous fall. Larvae that successfully feed in the summer or fall moult over winter and emerge as nymphs the following spring/summer; therefore, nymphs collected in the spring can be considered as belonging to the same cohort as the previous fall’s larvae. We therefore combined fall 2017 with spring 2018 data, and fall 2018 with spring 2019 data, and applied the indicator to each successive cohort for a given site. If only one season of data was available (either fall or spring for a given cohort), the indicator was applied using only the available season. For the 4 sites with only fall 2018 and spring 2019 data available, the indicator was applied once to the combined data for this cohort. One site, Petrie Island, had only one season of field sampling data available and the indicator was applied solely to this season.

The Clow et al. indicator was developed to be applied to a single drag session [13]. In order to apply it to combined data from multiple drags with varying amounts of person-hours of sampling, three alterations were made to the indicator flow chart. First, questions 1 (“what life stage did you collect?”) and 2 (“did you collect more than one *I*. *scapularis* of the same life stage?”) were applied to the combined data from both drags (i.e. sampling of one tick cohort). Second, question 3 was modified from “did you collect five or more *I*. *scapularis*?” to “was total *I*. *scapularis* density greater than or equal to 1.6667 ticks per person-hour?”. This value was determined by dividing five ticks by 3 person-hours, which was the sampling period used by Clow et al. to create and evaluate the indicator [13]. Finally, question 4 was adjusted to be “have *I*. *scapularis* been collected by dragging at this site *prior to this cohort*?”, rather than “have *I*. *scapularis* ben collected by dragging at this site before?”.

### Indicator performance

We assessed the consistency of risk indicator classification between two successive tick cohorts, denoted herein as indicator performance, following the method described by Clow et al. [13]. The kappa statistic was used to determine the degree of agreement between the results from the field samplings at the 23 sites for which two cohorts of data were available. Both an unweighted and weighted kappa were calculated [26]. The weighted kappa takes into account the degree of agreement between adjacent levels, which decreases as the difference between levels increases. This was done to allow for changing levels over time, as would be expected in a region of recent and ongoing tick population establishment [4, 13]. A quadratic weighting system was used, assigning 1.0 for perfect agreement, 0.8889 for partial agreement (one level removed), 0.5556 for limited agreement (two levels removed) and 0.000 for no agreement (three levels removed). The kappa value was interpreted using the guidelines established by Landis and Koch [27]. The *vcd* package in R 3.6.1 was used to calculate the kappa statistics and 95% confidence intervals, with a significance level of α = 0.05 [28, 29].

### Ecological classification

The 23 sites to which the risk indicator was applied were assigned an ecological classification based on their risk for *I*. *scapularis* establishment. While the risk level provides a useful guide for public health decision-making in a given year, an ecological classification describes the pattern of tick colonization over two successive cohorts. Initial colonization by *I*. *scapularis* may be followed by either establishment of a reproducing population or stochastic fade-out [4]. In addition, *I*. *scapularis* invasion in eastern Ontario generally precedes widespread infection with *B*. *burgdorferi* [11]. Therefore, an ecological classification that is based on two successive cohorts can provide additional information on the probability that a given site has an endemic population. Furthermore, comparing this classification with *B*. *burgdorferi* infection rates can indicate the risk of LD transmission in a given area.

Sites that were high risk in both cohorts, or medium risk in one and high risk in the other, were designated as “high-stable”, indicating the presence of a reproducing population of *I*. *scapularis*. Sites that were medium risk in both cohorts, or medium risk in one and low risk in the other, were designated “emerging”, indicating that a reproducing population of ticks may be developing, although a risk of stochastic fade-out still remains. Sites with a non-zero risk in one cohort and low risk in the other were designated “low-stable”, indicating that while the presence of *I*. *scapularis* has been detected, there is not yet evidence of a reproducing tick population. Sites with a non-zero risk in both cohorts carried though this non-zero terminology to represent the possibility of adventitious *I*. *scapularis* from migratory birds [13].

## Results

### Field sampling

We collected a total of 782 *I*. *scapularis* ticks over 317.49 person-hours of field sampling including 603 adults, 132 nymphs, and 47 larvae. Four additional species of tick were collected across five sites and two years–*Haemaphysalis leporispalustris* (n = 5) at one site in 2017 and three sites in 2018, *Ixodes marxi* (n = 1) at one site in 2017, *Ixodes muris* (n = 1) at one site in 2018, and *Ixodes cookei* (n = 1) at one site in 2018. Total annual site-specific *I*. *scapularis* density ranged from 0 to 16.3 ticks per person-hour (Table 1), with an overall three-year density of 2.46 ticks per person-hour. Mean *I*. *scapularis* density was 2.7 (standard deviation [SD] 5.4) in 2017, 1.8 (3.5) in 2018, and 3.5 (3.6) in 2019. Nymphal *I*. *scapularis* density ranged from 0 to 11.3 nymphs per person-hour, with an overall three-year density of 0.42 nymphs per person-hour. Mean (SD) nymphal density was 0.1 (0.3) in 2017, 0.1 (0.3) in 2018, and 1.1 (2.6) in 2019.

**Table 1:** Abundance and density of *Ixodes scapularis* ticks collected by drag sampling at Ottawa, Ontario sites and the site-specific prevalence of infection with *Borrelia burgdorferi*, 2017–2019.

Site ID	Site Name	Year	Person-Hours	Abundance			Densities		Annual Bb Infection Rate (%)		Pooled* Bb Infection Rate (%)	
				Larva	Nymph	Adult	Nymph	All Life Stages	Adult	Nymph	Adult	Nymph
1	Britannia Conservation Area	2017	8.35	0	0	15	0	1.80	13.33	-	17.65	-
		2018	6.00	0	0	1	0	0.17	100.00	-		
		2019	2.00	0	0	1	0	0.50	0	-		
3	Rideau River Eastern Pathway	2017	6.00	0	0	0	0	0	-	-	-	-
		2018	6.00	0	0	0	0	0	-	-		
		2019	2.00	0	0	0	0	0	-	-		
4	Beryl Gaffney Park	2017	6.57	0	0	1	0	0.15	0	-	0	-
		2018	6.00	0	0	1	0	0.17	0	-		
		2019	2.00	0	0	0	0	0	-	-		
5	Dominion Arboretum	2017	6.95	0	0	0	0	0	-	-	-	-
		2018	6.00	0	0	0	0	0	-	-		
		2019	2.00	0	0	0	0	0	-	-		
6	Heritage Park	2017	7.65	0	0	1	0	0.13	0	-	0	-
		2018	6.00	0	0	0	0	0	-	-		
		2019	2.00	0	0	0	0	0	-	-		
7	Greenbelt Pathway West	2017	7.96	1	6	10	0.75	2.14	40.00	16.67	26.09	5.26
		2018	6.00	0	1	22	1.67	3.83	22.73	0		
		2019	3.00	0	12	14	4.00	8.67	21.43	0		
8	Pine Grove	2017	8.25	3	0	0	0	0.36	-	-	-	-
		2018	6.00	0	0	0	0	0	-	-		
		2019	-	-	-	-	-	-	-	-		
9	South March Conservation Forest	2017	7.24	0	2	23	0.28	3.45	34.78	0	38.61	40.00
		2018	6.00	0	0	38	0.00	6.33	34.21	-		
		2019	3.00	0	8	40	2.67	16.00	45.00	50.00		
10	Morris Island Conservation Area	2017	8.50	0	4	25	0.47	3.41	40.00	0	35.48	10.52
		2018	-	-	-	-	-	-	-	-		
		2019	3.00	9	34	6	11.33	16.33	16.67	11.76		
11	Stoney Swamp	2017	13.23	4	11	11	0.83	1.97	63.64	0	47.73	0
		2018	6.00	1	2	16	0.33	3.17	25.00	0		
		2019	3.00	0	0	17	0	5.67	58.82	-		
12	Petrie Island	2017	2.58	0	0	12	0	4.65	8.33	-	8.33	-
		2018	-	-	-	-	-	-	-	-		
		2019	2.00	0	0	0	0	0	-	-		
15	Meadowbrook Park	2017	3.80	0	0	0	0	0	-	-	-	-
		2018	3.50	0	0	0	0	0	-	-		
		2019	2.00	0	0	0	0	0	-	-		
16	Prescott & Russell Recreational Trail	2017	6.25	0	0	0	0	0	-	-	12.50	-
		2018	6.00	0	0	8	0	1.33	12.50	-		
		2019	2.00	0	0	0	0	0	-	-		
17	Pinhey’s Point Park	2017	5.65	11	0	8	0	3.36	50.00	-	42.56	4.76
		2018	6.00	0	0	19	0	3.17	36.84	-		
		2019	3.00	18	21	1	7.00	13.33	100	4.76		
18	Brown’s Inlet Park	2017	4.25	0	0	0	0	0	-	-	-	-
		2018	3.50	0	0	0	0	0	-	-		
		2019	2.00	0	0	0	0	0	-	-		
19	Fairmont Park	2017	4.35	0	0	0	0	0	-	-	-	-
		2018	4.50	0	0	0	0	0	-	-		
		2019	2.00	0	0	0	0	0	-	-		
20	Carling Campus Northern Access	2017	4.00	0	0	6	0	1.50	50.00	-	61.90	-
		2018	6.00	0	0	1	0	0.17	0	-		
		2019	2.00	0	0	12	0	7.00	71.43	-		
21	Shirley’s Bay	2017	4.00	0	0	46	0	11.5	43.48	-	43.86	11.11
		2018	6.00	0	7	91	1.67	16.33	45.05	14.29		
		2019	3.00	0	2	34	0.67	12.00	41.18	0		
22	Beacon Hill	2017	1.16	0	0	15	0	12.93	13.33	-	10.00	0
		2018	6.00	0	0	4	0	0.67	0	-		
		2019	2.00	0	1	1	0.50	1.00	0	0		
23	Hog’s Back Park	2017	1.00	0	0	0	0	0	-	-	0	-
		2018	6.00	0	0	2	0	0.33	0	-		
		2019	2.00	0	0	1	0	0.50	0	-		
24	Carp Hill	2017	1.5	0	0	18	0	12.00	5.56	-	34.33	23.53
		2018	6.00	0	7	32	1.17	6.50	46.88	0		
		2019	3.00	0	10	17	3.33	9.00	41.18	40.00		
25	Greely/ Findlay Creek	2017	2.00	0	0	3	0	1.50	0	-	0	-
		2018	6.00	0	0	1	0	1.67	0	-		
		2019	2.00	0	0	1**	0	0.50	-	-		
26	Rockcliffe Park	2017	-	-	-	-	-	-	-	-	-	-
		2018	4.50	0	0	0	0	0	-	-		
		2019	2.00	0	0	0	0	0	-	-		
27	Mer Bleue Bog	2017	-	-	-	-	-	-	-	-	15.38	25.00
		2018	6.00	0	4	10	0.67	2.33	20.00	25.00		
		2019	2.00	0	0	3	0	1.50	0	-		
28	Brewer Park	2017	-	-	-	-	-	-	-	-	-	-
		2018	1.50	0	0	0	0	0	-	-		
		2019	2.00	0	0	0	0	0	-	-		
29	Strathcona Park	2017	-	-	-	-	-	-	-	-	-	-
		2018	1.50	0	0	0	0	0	-	-		
		2019	2.00	0	0	0	0	0	-	-		
30	Black Rapids Creek	2017	-	-	-	-	-	-	-	-	46.15	-
		2018	3.00	0	0	8	0	2.67	62.50	-		
		2019	3.00	0	0	5	0	1.67	20.00	-		
31	Ottawa River Pathway (Remic Rapids)	2017	-	-	-	-	-	-	-	-	-	-
		2018	1.50	0	0	0	0	0	-	-		
		2019	2.00	0	0	0	0	0	-	-		

Bb = *Borrelia burgdorferi*; Grey shading indicates sites that were not sampled in a given year;

*Pooled refers to stage-specific infection rate over all years of sampling;

**Specimen not available for testing

### Laboratory analyses

All 132 *I*. *scapularis* nymphs and 603 adults were tested for *B*. *burgdorferi*, *B*. *miyamotoi*, and *A*. *phagocytophilum*. The site-specific annual and pooled (i.e. combined over all sampling years) prevalence of *B*. *burgdorferi* in adult and nymphal *I*. *scapularis* ticks is shown in Table 1. In 2017, 11 of 22 sites sampled were found to have *I*. *scapularis* ticks infected with *B*. *burgdorferi* with 29.0% of adult and nymphal ticks testing positive. In 2018, 10 of 26 sites had *I*. *scapularis* ticks infected with *B*. *burgdorferi* with 34.9% of ticks testing positive. In 2019, 10 of 27 sites had *I*. *scapularis* ticks infected with *B*. *burgdorferi* and 32.2% of ticks tested positive. Overall, 12.1% of nymphal ticks were infected with *B*. *burgdorferi* compared to 36.7% of adult ticks. Differences in annual average infection rates may reflect the number, location, and timing of field samplings each year, which was dependent on logistics and weather. *Borrelia miyamotoi* was detected at 2 sites (sites 9 and 21) and in 0.005% (n = 3) of all ticks tested, and *A*. *phagocytophilum* was detected at 3 sites (sites 10, 17 and 21) and in 0.005% (n = 3) of all ticks tested. Although the focus of this study is on *B*. *burgdorferi*, results for other pathogens that were included in the testing algorithm are presented given their public health importance.

### Patterns in *I*. *scapularis* density and *B*. *burgdorferi* infection prevalence

**Fig 2:**
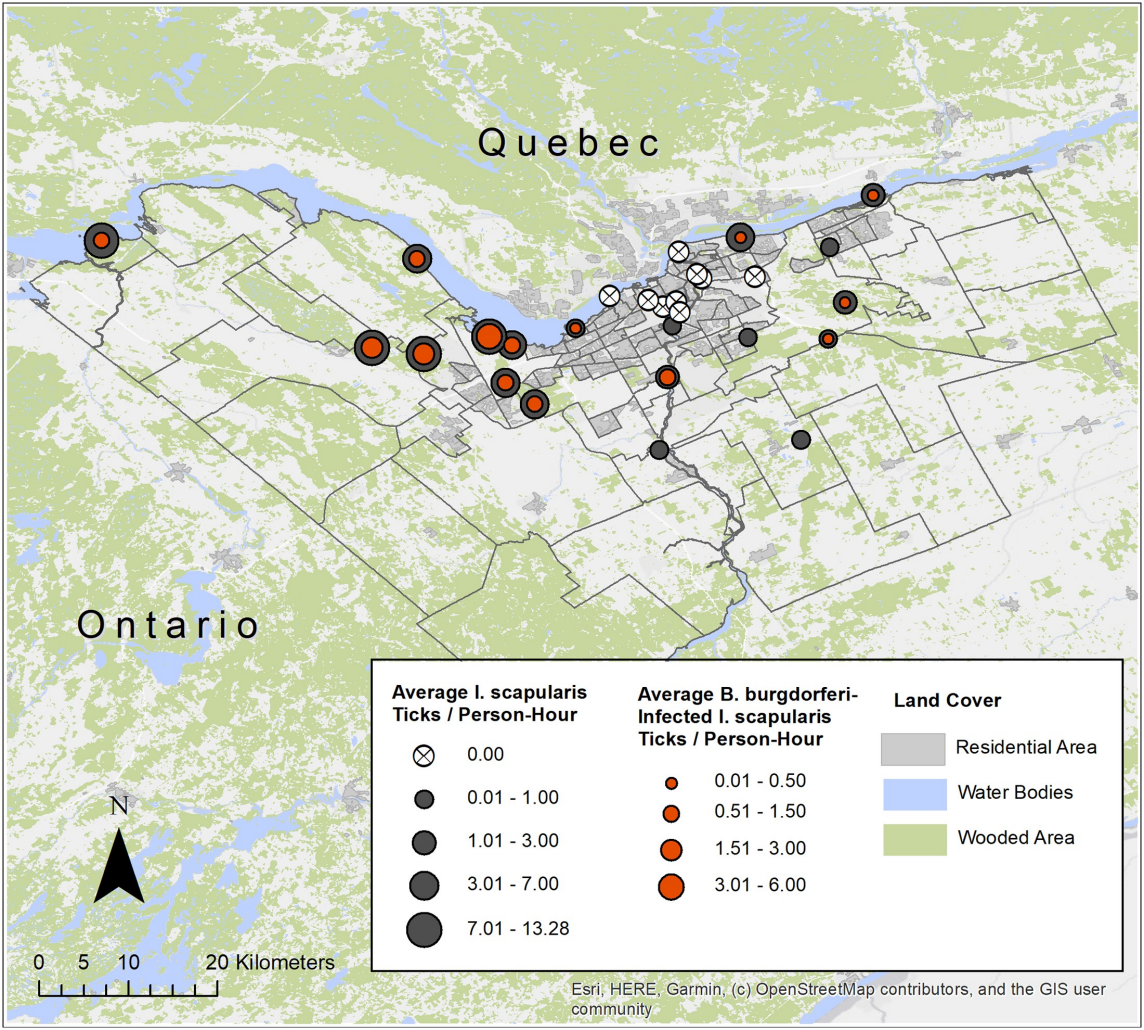
Map of total *Ixodes scapularis* density and the density of *Borrelia burgdorferi* infected ticks at 28 sampling sites in Ottawa, Ontario, 2017–2019.

Average *I*. *scapularis* density was greatest in sites located within the Greenbelt zone, in the suburban/rural areas of the western portion of the city of Ottawa, and along the Ottawa River, and generally lowest at sites in the suburban/urban core, similar to patterns previously described by Kulkarni et al. (Fig 2) [30]. The proportion of sites found to have ticks infected with *B*. *burgdorferi* varied by year and site, with an annual high of 50% of sites in 2017 and annual low of 37% in 2019. Infection rates were highest in sites located within and to the west of the Greenbelt zone, and along the Ottawa River, following roughly the same pattern observed for *I*. *scapularis* densities (Fig 2).

### Application of risk indicator

The risk indicator developed by Clow et al. was applied to determine the risk of *I*. *scapularis* establishment at each site. Between-cohort comparisons were made for the 23 sites for which two cohorts of sampling data were available. In the fall 2017 –spring 2018 cohort, 10 sites scored as high risk of *I*. *scapularis* establishment, 5 sites as medium risk, 2 sites as low risk, and 6 sites as non-zero (S1 Table). In the fall 2018 –spring 2019 cohort, 9 sites were high risk, 3 sites were medium risk, 5 sites were low risk, and 6 sites were non-zero. Sixteen sites maintained the same risk level between cohorts, 2 sites increased in risk by one level, and 5 sites decreased in risk by one level (S1 Table). Of the 4 sites sampled only during the second cohort (fall 2018 and spring 2019), 3 were designated non-zero risk and one was designated high risk (S1 Table). One site, Petrie Island, was only surveyed in the fall of 2017 and was designated high risk (S1 Table).

### Indicator performance

Indicator performance for 23 sites (i.e. the consistency of risk indicator classification between two successive tick cohorts) was measured using the kappa statistic. The unweighted kappa was 0.63 (95% CI 0.40–0.87) (p<0.001), which is within the range of values indicating substantial agreement. The weighted kappa was 0.92 (95% CI 0.84–0.99) (p<0.001), which is within the range of values for almost perfect agreement [27].

### Ecological classification

**Fig 3:**
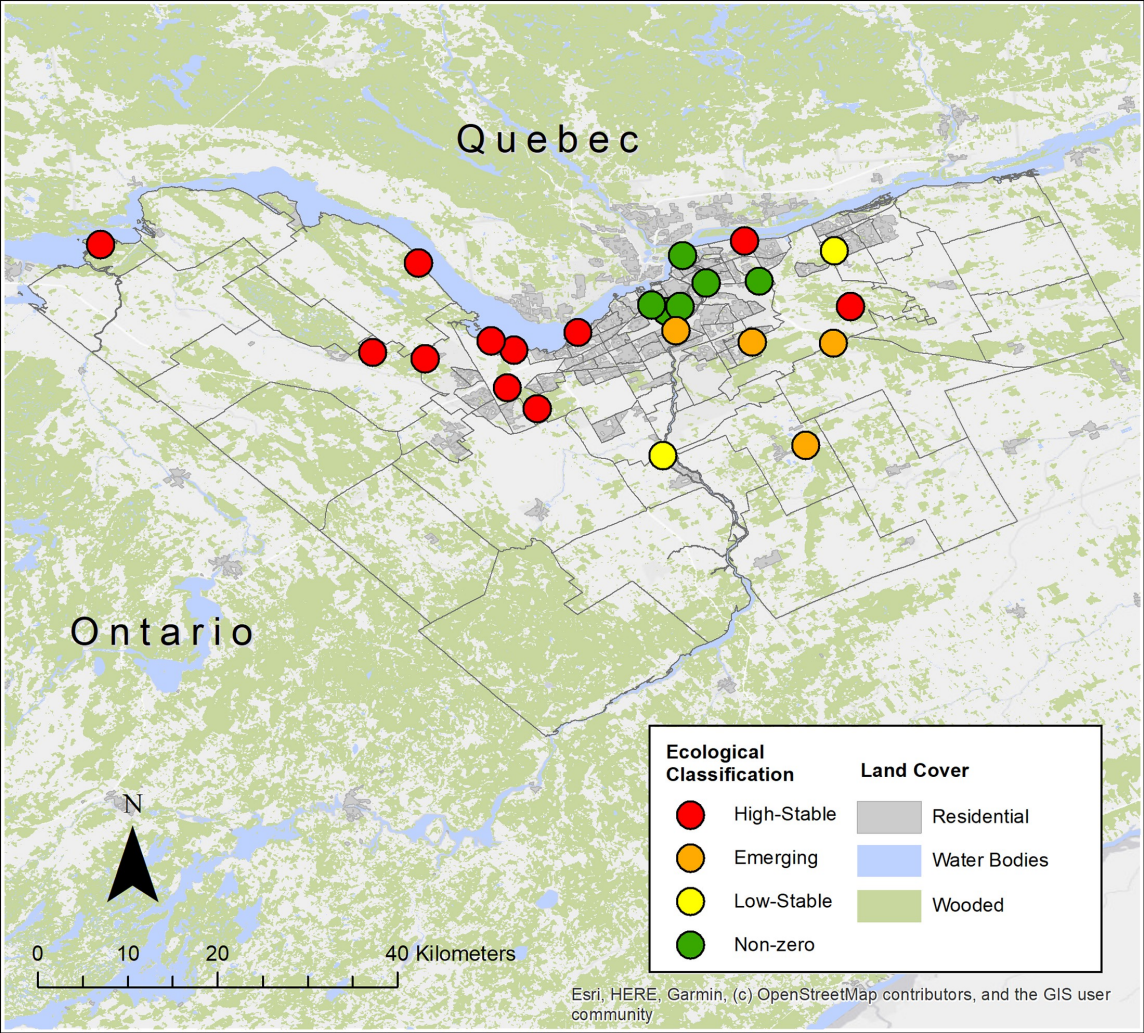
Ecological classification of tick sampling sites in Ottawa, Ontario, based on tick establishment risk indicator data from two successive cohorts of *Ixodes scapularis* ticks, 2017–2018 and 2018–2019.

Sites were assigned an ecological classification based on the results of the risk indicator assessment. Of the 23 sites for which data for two tick cohorts was available, 11 sites were classified as “high-stable”, 4 were classified as “emerging”, 2 were classified as “low-stable”, and 6 were classified as “non-zero” (Fig 3, S1 Table). All 11 high-stable sites had *B*. *burgdorferi* infected ticks, while only one cohort of one of the 4 emerging sites had infected ticks. *B*. *burgdorferi* was not detected in any of the low-stable or non-zero sites.

## Discussion

This study used active tick surveillance over multiple years to provide an up-to-date portrait of tick population establishment and LD risk areas in Ottawa, Ontario, a major urban centre at the edge of the northern range of tick establishment in eastern Canada [31]. Such a portrait is important for public health planning and interventions, and as a baseline for monitoring the ongoing invasion of ticks and their associated pathogens in Ontario. Our findings show that 68% of sampled sites were positive for *I*. *scapularis* ticks in at least one year, with 46% of sites positive in all years sampled. The prevalence of *B*. *burgdorferi* varied across sites ranging from 0–48% in adult ticks and 0–40% in nymphal ticks, reflecting geographic variation in human LD exposure risk. Importantly, this study revealed multiple areas with strong evidence of tick population establishment, mainly located in areas with large tracts of mixed/deciduous forest and/or wetlands, outside of the suburban/urban core. Many of these areas border residential neighbourhoods and/or constitute popular recreational areas for Ottawa residents, highlighting the risk for potential human exposure to ticks and tick-borne pathogens.

By applying a risk indicator to assess the likelihood of tick establishment, and comparing risk indicator assessments across two *I*. *scapularis* cohorts, we gained insight into the ecological state of *I*. *scapularis* population invasion in the Ottawa area. This approach provides more robust and complete picture of *I*. *scapularis* establishment than would be possible using the risk indicator for one drag or cohort alone. We found that all nine sites west of the Rideau River, i.e. those located along the Ottawa River, within the Greenbelt zone, and further west, are likely to have “high-stable” populations of *I*. *scapularis*. An additional two sites east of the Rideau River were found to have “high-stable” populations. Seven of these “high-stable” sites had *B*. *burgdorferi*-infected ticks in both cohorts, and the remaining two had infected ticks in one cohort. This finding suggests a stable LD risk at high-stable sites over time, although there were differences in tick densities and *B*. *burgdorferi* prevalence between sites. Four sites along and east of the Rideau River were deemed to have “emerging” *I*. *scapularis* tick populations, while the six sites closest to the urban/suburban core displayed a “non-zero” risk of established or emerging tick populations. Previous studies in the Ottawa area have demonstrated a positive association between the amount of forested area and *I*. *scapularis* density [17], while forest structure and composition have also been associated with *I*. *scapularis* occurrence and density [7, 17]. In particular, areas with deciduous or mixed forest favour tick establishment owing to the presence of suitable tick habitat and key wildlife host species, such as white-tailed deer [23]. The ‘high-stable’ sites identified in this study corresponded to rural or suburban areas with a high proportion of deciduous and mixed forest. In contrast, ‘emerging’ sites were generally situated in areas with a lower forested area, and higher proportion of the surrounding area comprised of built-up or agricultural landuse. Further investigation and characterization of differences in landuse and landcover composition across the study area, including forest fragmentation, patch size and connectivity at different spatial scales, are needed to identify the factors driving tick population establishment in the city of Ottawa and surrounding areas, and the potential impacts of urban development.

Given that the risk indicator is a relatively new tool for determining the likelihood of *I*. *scapularis* establishment, we assessed whether it was performing as expected when applied to a new field sampling location and examined its consistency over two tick cohorts. The weighted kappa value for the degree of agreement between the 2017–2018 cohort and the 2018–2019 cohort was found to be within the range of almost perfect agreement, suggesting that the indicator is performing reliably over time, and that little ecological change has occurred over the study period. We expected that any changes in risk levels between cohorts would be increases, as we anticipate ongoing tick range expansion and/or establishment [13]. However, we found that five sites decreased by one risk level while only one site increased in risk. This may be due to variations in the timing of sampling between cohorts and/or stochastic fadeout of new tick populations. For sites that changed from medium risk in one cohort to low risk in the next, future studies could help to determine whether stochastic fadeout is occurring and the frequency with which this occurs. We adapted the tool to be used for multiple field samplings in the same cohort by making slight adjustments to the questions asked. A benefit of this approach is that it allows more data to be considered when determining a risk level, improving the likelihood of a representative assessment. However, it is possible that if we had applied the tool in its original form—that is, to only one field sampling—we would have acquired more conservative risk assessments due to the more limited opportunity to collect multiple tick life stages, or more than one tick of the same life stage.

Although we combined the results of two field samplings into one cohort to conduct analyses, the number of ticks tested from each site and cohort was often small (n<30), reducing the robustness of pathogen prevalence estimates; these estimates should therefore be interpreted with caution. The low infection rates found in some high tick-density sites may be due to variation in the lag time between tick population establishment and *B*. *burgdorferi* establishment, or to inter-annual fluctuations in host abundance and/or the proportion of tick blood meals coming from different host species [11, 32, 33]. Conversely, the high infection rates found in some low-density sites may reflect the small sample size of ticks collected. The timing of field sampling varied between years due to weather and logistical factors (for example, flooding), and not every site was sampled every year. This may impact both the total number of ticks collected and the proportion belonging to each life stage. The phenology of ticks is such that peak adult density occurs in the fall and subsequent spring, while peak nymphal density occurs in the summer [13, 23, 34]. While a decreasing abundance of the different life stages is generally expected from larvae to nymphs to adults, we observed higher adult abundance relative to nymphs, and nymphs relative to larvae, because of the timing of the sample collections. Detection of ticks in areas of very recent tick establishment may have been limited by the drag sampling method, which is only 50% sensitive (when compared to the gold standard that involves resource-intensive small mammal sampling) and presents a risk for false negatives in low-density populations [9]. Surveillance efforts in this study were targeted to sentinel locations owing to time and resource constraints; while the sites cover a broad geographic area, other areas with established tick populations are likely to exist that were not identified in this study.

All sites surveyed were parks, recreational trails, and conservation areas that are in or near residential areas; these are places frequented by local residents and visitors for leisure and exercise. Therefore, our findings have important implications for public health communication and interventions. Sites ecologically categorized as “high-stable” pose a risk of LD exposure to the community as they have reproducing tick populations with low-to-moderate levels of *B*. *burgdorferi* infection. While these sites do not constitute the only areas of potential Lyme disease risk, they can be targeted for public communication and education by public health and/or conservation authorities alongside more general risk communication regarding habitats that would present higher risk. This includes encouraging residents and visitors to wear long pants, use insect repellent, or wear clothing impregnated with permethrin and engage in self-examination to allow prompt tick removal if necessary. In addition, these sites may be considered for additional interventions such as woodchip barriers and vegetation trimming along trails, the removal of leaf litter, and lawn mowing [24, 35], although the effectiveness of these interventions in a local context requires further evaluation. “Emerging” sites, of which one had ticks infected with *B*. *burgdorferi*, should also be considered for the initiation of public health interventions and prioritized for future active surveillance. Low risk sites should be reassessed in succeeding cohorts [13]. The ecological classification of sites based on multi-year surveillance efforts can help to prioritize yearly surveillance efforts to “emerging” and “low” risk areas, as well as new areas of potential future tick establishment as indicated by predictive models (e.g. [7, 8]), with periodic assessment of “high-stable” sites to monitor longer-term trends.

With ongoing climate change, there is a need for continued surveillance for *I*. *scapularis* and *B*. *burgdorferi* as well as other emerging tick species and tick-borne pathogens. In addition to *I*. *scapularis*, we detected four other tick species including *H*. *leporispalustris*; this species is known to carry the zoonotic pathogens *Rickettsia rickettsii*, the causative agent of Rocky Mountain Spotted Fever, and *Francisella tularensis*, the causative agent of tularemia [36]. These pathogens are also known to be carried by the Lone Star tick, *Amblyomma americanum*, which is expected to benefit from climate change-induced range expansion into eastern Ontario [37]. In *I*. *scapularis* ticks, we detected a low prevalence of both *A*. *phagocytophilum* and *B*. *miyamotoi*, which cause anaplasmosis and relapsing fever, respectively. Continued surveillance for both *I*. *scapularis* and *B*. *burgdorferi*, and other emerging tick species and pathogens, is particularly important in the city of Ottawa where population growth and urban expansion are putting more people into contact with established tick populations, increasing the risk of infection with LD.

## Supporting information

S1 Table Scoring of tick sampling sites in Ottawa, Ontario over two successive tick cohorts, 2017–2018 and 2018–2019, using the Clow et al. indicator to assess the risk of tick establishment, and the resulting site-specific ecological classification. (DOCX)
