# Supplementary material for: A multi-year assessment of blacklegged tick (Ixodes scapularis) population establishment and Lyme disease risk areas in Ottawa, Canada, 2017-2019
Source: PLoS One. 2021 Feb 4;16(2):e0246484. doi: 10.1371/journal.pone.0246484 (PMC7861446; doi:10.1371/journal.pone.0246484)
Supplement: S1 Table — (DOCX) [file pone.0246484.s001.docx]

**S1 Table.** Scoring of tick sampling sites in Ottawa, Ontario over two successive tick cohorts, 2017-2018 and 2018-2019, using the Clow et al. indicator to assess the risk of tick establishment, and the resulting site-specific ecological classification.

| Site ID | Site Name | Cohort | | Person-Hours of Sampling | Abundance | | | Total Density | Total Points | Risk Level | Ecological Classification |
| --- | --- | --- | --- | --- | --- | --- | --- | --- | --- | --- | --- |
|  |  | Year | Season |  | Larva | Nymph | Adult |  |  |  |  |
| 1 | Britannia Conservation Area | 2017-2018 | Fall-Spring | 7.4 | 0 | 0 | 15 | 1.97 | 5 | High | High-Stable |
|  |  | 2018-2019 | Fall-Spring | 4 | 0 | 0 | 2 | 0.50 | 4 | Medium |  |
| 3 | Rideau River Eastern Pathway | 2017-2018 | Fall-Spring | 6 | 0 | 0 | 0 | 0 | 0 | Non-Zero | Non-Zero |
|  |  | 2018-2019 | Fall-Spring | 5 | 0 | 0 | 0 | 0 | 0 | Non-Zero |  |
| 4 | Beryl Gaffney Park | 2017-2018 | Fall-Spring | 9.57 | 0 | 0 | 1 | 0 | 1 | Low | Low-Stable |
|  |  | 2018-2019 | Fall-Spring | 5 | 0 | 0 | 1 | 0 | 1 | Low |  |
| 5 | Dominion Arboretum | 2017-2018 | Fall-Spring | 6.35 | 0 | 0 | 0 | 0 | 0 | Non-Zero | Non-Zero |
|  |  | 2018-2019 | Fall-Spring | 5 | 0 | 0 | 0 | 0 | 0 | Non-Zero |  |
| 6 | Heritage Park | 2017-2018 | Fall-Spring | 6.85 | 0 | 0 | 0 | 0 | 1 | Low | Low-Stable |
|  |  | 2018-2019 | Fall-Spring | 5 | 0 | 0 | 0 | 0 | 1 | Low |  |
| 7 | Greenbelt Pathway West | 2017-2018 | Fall-Spring | 6.61 | 1 | 4 | 11 | 2.42 | 10 | High | High-Stable |
|  |  | 2018-2019 | Fall-Spring | 9 | 0 | 13 | 29 | 4.67 | 8 | High |  |
| 8 | Pine Grove | 2017-2018 | Fall-Spring | 7.25 | 0 | 0 | 3 | 0.41 | 4 | Medium | Emerging |
|  |  | 2018 | Fall | 3 | 0 | 0 | 0 | 0 | 1 | Low |  |
| 9 | South March Conservation Forest | 2017-2018 | Fall-Spring | 7.13 | 0 | 0 | 46 | 6.45 | 6 | High | High-Stable |
|  |  | 2018-2019 | Fall-Spring | 6 | 0 | 8 | 52 | 10.00 | 8 | High |  |
| 10 | Morris Island Conservation Area | 2017 | Fall | 4.45 | 0 | 0 | 21 | 4.72 | 6 | High | High-Stable |
|  |  | 2019 | Spring | 3 | 9 | 34 | 6 | 16.33 | 10 | High |  |
| 11 | Stoney Swamp | 2017-2018 | Fall-Spring | 11.53 | 5 | 9 | 16 | 2.60 | 10 | High | High-Stable |
|  |  | 2018-2019 | Fall-Spring | 6 | 0 | 1 | 22 | 3.83 | 8 | High |  |
| 12 | Petrie Island | 2017 | Fall | 2.58 | 0 | 0 | 12 | 4.65 | 5 | High | N/A |
| 15 | Meadowbrook Park | 2017-2018 | Fall-Spring | 3.65 | 0 | 0 | 0 | 0 | 0 | Non-Zero | Non-Zero |
|  |  | 2018-2019 | Fall-Spring | 3.50 | 0 | 0 | 0 | 0 | 0 | Non-Zero |  |
| 16 | Prescott & Russell Recreational Trail | 2017-2018 | Fall-Spring | 5.3 | 0 | 0 | 8 | 1.51 | 3 | Medium | Emerging |
|  |  | 2018-2019 | Fall-Spring | 5 | 0 | 0 | 0 | 0 | 1 | Low |  |
| 17 | Pinhey’s Point Park | 2017-2018 | Fall-Spring | 6 | 11 | 0 | 18 | 4.83 | 7 | High | High-Stable |
|  |  | 2018-2019 | Fall-Spring | 6 | 18 | 21 | 10 | 8.17 | 10 | High |  |
| 18 | Brown’s Inlet Park | 2017-2018 | Fall-Spring | 3.8 | 0 | 0 | 0 | 0 | 0 | Non-Zero | Non-Zero |
|  |  | 2018-2019 | Fall-Spring | 3.5 | 0 | 0 | 0 | 0 | 0 | Non-Zero |  |
| 19 | Fairmont Park | 2017-2018 | Fall-Spring | 4.65 | 0 | 0 | 0 | 0 | 0 | Non-Zero | Non-Zero |
|  |  | 2018-2019 | Fall-Spring | 3.5 | 0 | 0 | 0 | 0 | 0 | Non-Zero |  |
| 20 | Carling Campus Northern Access | 2017-2018 | Fall-Spring | 7 | 0 | 0 | 6 | 0.86 | 3 | Medium | Emerging |
|  |  | 2018-2019 | Fall-Spring | 5 | 0 | 0 | 15 | 3.00 | 6 | High |  |
| 21 | Shirley’s Bay | 2017-2018 | Fall-Spring | 7 | 0 | 7 | 81 | 12.57 | 7 | High | High-Stable |
|  |  | 2018-2019 | Fall-Spring | 6 | 0 | 2 | 90 | 15.33 | 8 | High |  |
| 22 | Beacon Hill | 2017-2018 | Fall-Spring | 4.16 | 0 | 0 | 17 | 4.09 | 5 | High | High-Stable |
|  |  | 2018-2019 | Fall-Spring | 5 | 0 | 1 | 3 | 0.8 | 6 | High |  |
| 23 | Hog’s Back Park | 2017-2018 | Fall-Spring | 4 | 0 | 0 | 2 | 0.5 | 3 | Medium | Emerging |
|  |  | 2018-2019 | Fall-Spring | 5 | 0 | 0 | 1 | 0.2 | 2 | Low |  |
| 24 | Carp Hill | 2017-2018 | Fall-Spring | 4.5 | 0 | 7 | 30 | 8.22 | 7 | High | High-Stable |
|  |  | 2018-2019 | Fall-Spring | 6 | 0 | 10 | 37 | 7.83 | 8 | High |  |
| 25 | Greely/  Findlay Creek | 2017-2018 | Fall-Spring | 5 | 0 | 0 | 3 | 0.6 | 3 | Medium | Emerging |
|  |  | 2018-2019 | Fall-Spring | 5 | 0 | 0 | 2 | 0.4 | 4 | Medium |  |
| 26 | Rockcliffe Park | 2018 | Spring | 3 | 0 | 0 | 0 | 0 | 0 | Non-Zero | Non-Zero |
|  |  | 2018-2019 | Fall-Spring | 3.5 | 0 | 0 | 0 | 0 | 0 | Non-Zero |  |
| 27 | Mer Bleue Bog | 2018 | Spring | 3 | 0 | 4 | 9 | 4.33 | 7 | High | High-Stable |
|  |  | 2018-2019 | Fall-Spring | 5 | 0 | 0 | 4 | 0.8 | 4 | Medium |  |
| 28 | Brewer Park | 2018-2019 | Fall-Spring | 3.5 | 0 | 0 | 0 | 0 | 0 | Non-Zero | N/A |
| 29 | Strathcona Park | 2018-2019 | Fall-Spring | 3.5 | 0 | 0 | 0 | 0 | 0 | Non-Zero | N/A |
| 30 | Black Rapids Creek | 2018-2019 | Fall-Spring | 6 | 0 | 0 | 13 | 2.17 | 6 | High | N/A |
| 31 | Ottawa River Pathway (Remic Rapids) | 2018-2019 | Fall-Spring | 3.5 | 0 | 0 | 0 | 0 | 0 | Non-Zero | N/A |

N/A = not assessed because risk indicator data only available for a single cohort
